# Optimized border irrigation improved soil water content, increased winter wheat grain yield and water productivity

**DOI:** 10.1038/s41598-022-25137-x

**Published:** 2022-11-29

**Authors:** Feilong Yan, Zhenwen Yu, Yu Shi

**Affiliations:** National Key Laboratory of Crop Biology, Agronomy College of Shandong Agricultural University, Tai’an, 271018 Shandong China

**Keywords:** Plant sciences, Photosynthesis, Plant physiology

## Abstract

Border irrigation is still the main irrigation method in the Huang-Huai-Hai Plain of China (HPC), we aimed to find a suitable border length to reduce the quantity of irrigation water through a traditional border irrigation system to alleviate groundwater depletion. A 2-year experiment (2017–2019) was conducted with four border lengths: 20 m (L20), 30 m (L30), 40 m (L40) and 50 m (L50); supplementary irrigation was implemented during jointing and anthesis. The results showed that compared with the L20 and L30 treatments, the L40 treatment did not significantly increase the total water consumption. Compared with the L50 treatment, the L40 treatment significantly reduced the water consumption of ineffective tillers from jointing to anthesis. There was no significant difference in flag leaf net photosynthetic rate (Pn) between L40 treatment and L50 treatment at 14–28 days after anthesis, which was 12.36% and 21.31% higher than L30 and L20 treatments respectively, and significantly increased dry matter accumulation after anthesis. Grain yield were the higher in the L40 and L50 treatments, while the water productivity (WP) was highest in the L40 treatment, which was 3.98%, 4.54% and 7.94% higher than L50, L30, and L20 treatments, respectively. Hence, the irrigation field treatments with a border length of 40 m were considered the most efficient, which provides a theoretical basis for optimizing the traditional irrigation border length in HPC.

## Introduction

The production of China’s wheat in 2020 was approximately 134 million tons, of which more than 60% originated from the Huang-Huai-Hai Plain of China (HPC); however, water resources in this area accounted for only 7% of China’s total^1,2^. Because of the monsoon climate affecting this region, there is less rainfall (100–180 mm) during the wheat growing season, which is insufficient to meet the water requirements of wheat (400–500 mm) in this region^3,4^. Therefore, supplementary irrigation is the main method to ensure a stable and high yield of wheat^5^. At present, traditional border irrigation is still the principal irrigation method used in the HPC^6^. Studies show that when the border length was 80–100 m, the single irrigation amount was approximately 100–150 mm, which far exceeds the water availability required for wheat growth^7^. Thus, excessive border length leads to excessive irrigation and consequently a significant reduction in water productivity^8^. However, Cui et al.^9^ surveyed approximately 300 plots in Huimin County, Shandong Province, and revealed that the border lengths of 87% of the irrigation fields were longer than 100 m. Moreover, we have also investigated the towns and villages where these experimental plots are located and our results showed that more than 97% of the plots have a border length of over 60 m, with some being even more than 200 m. Therefore, a field experiment is needed to determine the appropriate border length of irrigated fields to improve the efficiency of irrigation water use.

More than 50% of the grain yield of wheat is owed to the accumulation of photosynthetic products after anthesis, and the soil water condition can significantly affect the accumulation of dry matter^10^. Drought after anthesis will have a negative effect on photosynthesis by shortening its duration and reducing the accumulation of photosynthetic product^11^. Indeed, a treatment of 70–75% soil water content showed a significantly higher Pn of flag leaves after anthesis, as well as an increase in dry matter accumulation, compared to a treatment of 50–55% soil water content. Water stress conditions can promote wheat grain filling and increase the dry matter accumulation during maturity, while excessive irrigation can reduce the distribution of dry matter in the grains after anthesis, thereby reducing the grain yield^12,13^. Therefore, it is important to study the effects of different border length irrigation on soil water content and dry matter accumulation and transport to determine the appropriate border length.

The objectives of the experiment are to (1) compare the soil water content in the 0–80 cm soil layer after irrigation with different border lengths, (2) investigate the differences in dry matter accumulation and transportation and grain yield with different border lengths, (3) clarify the relationship between soil water content in the 0–80 cm soil layer after irrigation and grain yield and WP.

## Materials and methods

### Experimental site

**Figure 1:**
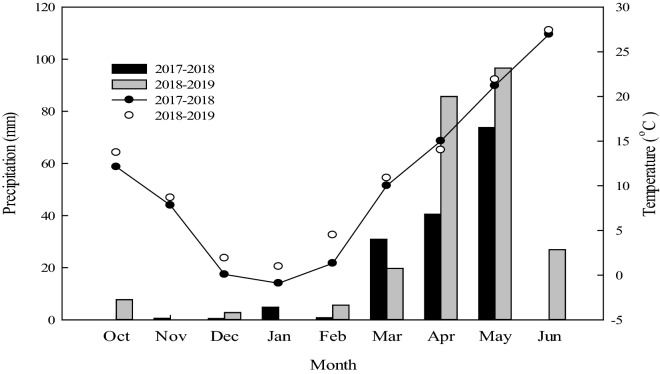
Precipitation and temperature during wheat growth period in 2017–2018 and 2018–2019.

In the 2017–2019 winter wheat growing seasons, a field experiment was carried out at the experimental station of Shijiawangzi village, Shandong Province, China (35° 42′ N, 116° 41′ E), which experiences a warm temperature continental climate. The soil in the region is classified as loam and composed of 29.6% clay, 37.3% silt and 33.1% sand. And Table 1 shows the nutrient content in the 0–20 cm soil layer, and Fig. 1 shows the precipitation and temperature at different months of wheat growth in this experiment. The soil bulk density and field capacity of the 0–200 cm soil layers of the experiment field are shown in Table 2.

**Table 1:** The nutrient content in the 0–20 cm soil layer before sowing.

Items	Growing season	
	2017–2018	2018–2019
Soil organic matter (g kg^−1^)	14.31	14.24
Total nitrogen (g kg^−1^)	1.17	1.09
Available nitrogen (mg kg^−1^)	118.82	117.32
Available phosphorus (mg kg^−1^)	39.29	36.71
Available potassium (mg kg^−1^)	116.37	122.18

**Table 2:** Soil bulk density and field capacity of 0–200 cm soil layers of the experiment field.

Soil layer	2017–2018		2018–2019	
	Soil bulk density	Field capacity	Soil bulk density	Field capacity
cm	g cm^−3^	%	g cm^−3^	%
0–20	1.46	28.22	1.41	29.45
20–40	1.58	24.53	1.57	24.08
40–60	1.56	25.17	1.54	25.74
60–80	1.58	24.31	1.58	23.94
80–100	1.59	24.22	1.61	23.42
100–120	1.57	23.98	1.58	24.11
120–140	1.57	24.75	1.56	25.03
140–160	1.56	23.38	1.57	23.86
160–180	1.57	24.87	1.56	24.27
180–200	1.56	24.79	1.56	24.61

### Experimental design and crop management

During the wheat growing seasons from 2017 to 2019, irrigation fields with three different border lengths were set up (border width, 2 m): 20 m (L20), 30 m (L30), and 40 m (L40) in 2017–2018 and 30 m (L30), 40 m (L40), 50 m (L50) in 2018–2019, and both had a control treatment without irrigation (RF). The treatments were randomly grouped and each treatment had three replicates. A 2-m-wide guard row was used to prevent water permeating between two adjacent irrigation plots. All treatments were irrigated from the same side of the field during the jointing and anthesis stages. Inflow cutoff was designed at 90% (that is, when irrigation is stopped when the waterfront reaches 90% of the border length), and the irrigation amount was measured by a flow meter^14^. The groundwater depth is about 25 m. The water output of the well in the experiment site was 30 m^3^ per hour and the amount of irrigation in the two growing seasons is shown in Table 3.

**Table 3:** The amount of irrigation for the different treatments.

Year	Treatment	Jointing (mm)	Anthesis (mm)	Total (mm)
2017–2018	RF	–	–	–
	L20	65.22	54.62	119.84
	L30	77.83	61.17	139
	L40	86.59	69.38	155.97
2018–2019	RF	–	–	–
	L30	63.17	58.11	121.28
	L40	73.02	68.05	141.07
	L50	85.27	79.11	164.38

The high-yielding wheat variety ‘Jimai 22’, the most widely cultivated commercial variety in the HPC, was used for this experiment. N 105 kg ha^−1^, P_2_O_5_ 150 kg ha^−1^, and K_2_SO_4_ 150 kg ha^−1^ were applied as basal fertilizers on all fields before sowing, and topdressing of N 135 kg ha^−1^ was applied at the jointing stage. The wheat was sown on October 20, 2017, and October 8, 2018, with planting densities of 2.7 million ha^−1^ and 1.8 million ha^−1^, respectively, and harvested on June 7, 2018, and June 12, 2019. No pests and diseases occurred during the test period.

### Sample and data collection

#### Sampling point

Divide the border length into an interval every 10 m, and take samples at the center of each interval. The test results are the measured values of the mixed samples at each sampling point under this treatment.

#### Soil water content

Soil samples were collected using a soil auger with 20 cm increments up to a depth of 200 cm before sowing and at the jointing, anthesis, maturity and 3 days after irrigation in all points. The soil water content was measured by the oven-drying method^15^.

#### Net photosynthetic rate

The Pn of the flag leaves were measured from 09:00 to 11:00 AM at anthesis and 7, 14, 21 and 28 days after anthesis (DAA) by Li-6400XT portable photosynthetic apparatus (LI-COR, Lincoln Nebraska, USA).

#### Population dynamics and dry matter accumulation

The number of tillers (stems) per square meter was investigated at jointing, 10 and 20 days after jointing, anthesis and maturity. At the anthesis and maturity stages, 50 plants of wheat accumulated on the ground were collected from each point. Samples were separated into stem, leaf, spike (spike axis and kernel husks) and grain (only at maturity). All plant samples were dried at 70 °C to a constant weight for determination of their biomass. The dry matter translocation (DMT), dry matter accumulation after anthesis (DMAA), and their contribution to grain were calculated according to the method of Chu et al.^16^.

#### Grain yield, ET and WP

Grain yield was determined from a 3 m^2^ area from each field at the maturity stage. The soil water consumption was calculated by the soil water content during the sowing and maturity period. In this experiment station, groundwater recharge and runoff can be ignored. Crop water consumption (ET) was calculated using the following soil water balance equation^17^:

$${\text{ET }} = {\text{ irrigation }} + {\text{ precipitation }} + {\text{ soil water consumption}},$$

WP was defined as follows^18^:

$${\text{WP }} = {\text{ grain yield}}/{\text{ET}}.$$

### Statistical analysis

SPSS Statistics 22.0 software (IBM, Armonk, NY, USA) was used to analyze the data and the least significant difference test (α = 0.05) was used to compare the differences between the different treatments. All charts were generated using Sigmaplot 12.0 (Systat Software Inc., San Jose, CA, USA).

### Statement

“Jimai 22”, the winter wheat cultivar that we used in the present experiment, complied with international guidelines. We complied with the IUCN Policy Statement on Research Involving Species at risk of extinction and the Convention on the Trade in Endangered Species of Wild Fauna and Flora.

## Results

### Soil water content

The results obtained for the two growing seasons were consistent (Table 4). The soil water content in the 0–40 cm surface soil layer before irrigation during the anthesis slightly differed (2017–2018) or showed no significant difference (2018–2019), while the soil water content in the 0–80 cm soil layer increased significantly with the increase in border length. Moreover, a longer irrigation border length resulted in a higher soil water content in the 0–40 cm and 0–80 cm soil layers after irrigation.

**Table 4:** Soil water content (%) in different soil layer after irrigation under different treatments.

Year	Treatment	Jointing				Anthesis			
		Before irrigation		3 days after irrigation		Before irrigation		3 days after irrigation	
		0–40 cm	0–80 cm	0–40 cm	0–80 cm	0–40 cm	0–80 cm	0–40 cm	0–80 cm
2017–2018	RF	42.34	55.83	40.86d	54.16d	35.25c	44.81d	33.36d	43.25d
	L20	42.34	55.83	66.35c	65.23c	37.72b	50.95c	67.84c	63.25c
	L30	42.34	55.83	71.5b	69.84b	39.97a	52.47b	71.19b	68.54b
	L40	42.34	55.83	75.6a	72.21a	40.54a	55.58a	75.04a	72.61a
2018–2019	RF	44.31	59.57	43.28d	58.33d	37.85b	47.59c	36.09d	46.32d
	L30	44.31	59.57	71.18c	69.57c	41.93a	52.25b	68.62c	65.44c
	L40	44.31	59.57	75.58b	73.46b	42.55a	56.15a	73.99b	72.56b
	L50	44.31	59.57	81.08a	80.63a	43.19a	57.18a	82.19a	80.42a

Different letters indicate significant statistical differences between treatments (P < 0.05).

### Water consumption in different growth stages

The water consumption of winter wheat at different growth stages in the two growing seasons is consistent. The water consumption from the sowing to jointing stage was lower when the temperature is lower, and as the temperature rose, the growth and development of winter wheat as well as the water consumption from jointing to anthesis and anthesis to maturity increased significantly (Table 5). Compared with the irrigation treatment, the RF treatment significantly reduced the water consumption. In 2017–2018, there was no significant difference in water consumption at different stages and in the total water consumption of the L20, L30 and L40 treatments. However, in 2018–2019, the water consumption from jointing to anthesis and anthesis to maturity and total water consumption were the highest in L50, followed by L40 and then L30.

**Table 5:** The water consumption (mm) of winter wheat in different growth stages under different treatment.

Year	Treatment	Sowing to jointing	Jointing to anthesis	Anthesis to maturity	Total
2017–2018	RF	95.96	100.61b	120.17b	316.74b
	L20	95.96	131.89a	182.04a	409.89a
	L30	95.96	133.65a	186.26a	415.87a
	L40	95.96	133.05a	188.03a	417.04a
2018–2019	RF	105.4	102.61c	185.48c	393.49c
	L30	105.4	141.05b	233.06b	479.51b
	L40	105.4	143.36b	236.67ab	485.43b
	L50	105.4	155.73a	239.38a	500.51a

Different letters indicate significant statistical differences between treatments (P < 0.05).

### Population dynamics

**Figure 2:**
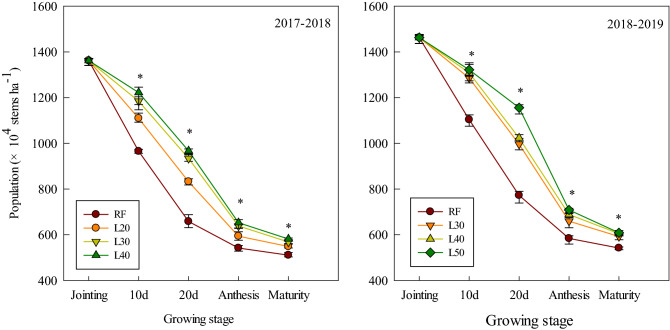
The population dynamics of winter wheat from jointing to maturity under different treatments. *Significant at the 0.05 probability level.

As shown in Fig. 2, the population of winter wheat declined rapidly from jointing to anthesis and the population of the RF treatment was significantly lower than that of the irrigation treatment at 10 days and 20 days after jointing, as well as in the anthesis and maturity stages. Compared with the L30 and L40 treatments, the L50 treatment significantly delayed the population number from 0 to 20 days after jointing, but after 20 days of jointing, the population number decreased rapidly. As the L20 treatment caused water stress, the population number from jointing to anthesis was decreased faster than that of the L30 and L40 treatments.

### Net photosynthetic rate

**Figure 3:**
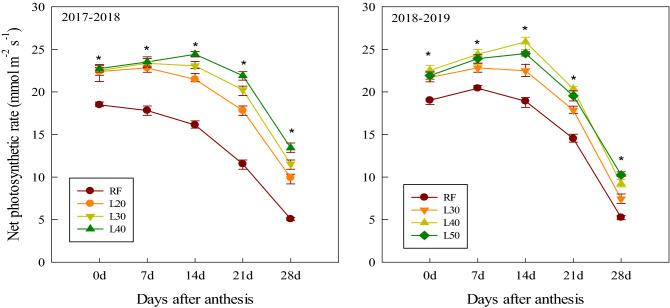
Net photosynthetic rate of different treatments in 2017–2018 and 2018–2019. *Significant at the 0.05 probability level.

Due to soil water stress after anthesis, the Pn of the flag leaves in the RF treatment was significantly lower than that of the other treatments in the two growing seasons (Fig. 3). Compared with L40 and L50, the Pn of L20 and L30 was significantly lower from 14 to 28 DAA. Moreover, there was no significant difference in Pn after anthesis between the L40 and L50 treatments, except that the L40 treatment was significantly higher than L50 treatment at 14 DAA.

### Dry matter accumulation and translocation

During the two growing seasons, the dry matter accumulation at anthesis and maturity of RF was significantly lower than that of the other treatments (Table 6). In the two growing seasons, there was no significant difference in the dry matter accumulation at anthesis of the border length treatment, while the DMAA, CDMAA and the dry matter accumulation at maturity (DMM) increased with the increase of the border length, and the difference between L40 and L50 was not significant. On the contrary, DMT and CDMT decreased significantly with the increase in border length.

**Table 6:** Dry matter accumulation amount at anthesis and maturity and dry matter translocation after anthesis under different treatments.

Year	Treatment	Dry matter accumulation amount (kg ha^−1^)		DMT	CDMT	DMAA	CDMAA
		Anthesis	Maturity	kg ha^−1^	%	kg ha^−1^	%
2017–2018	RF	9146.45b	12,050.81c	2459.82a	45.86a	2904.36d	54.14d
	L20	10,534.55a	15,357.60b	2475.88a	33.92b	4823.05c	66.08c
	L30	10,817.65a	16,053.15ab	2421.42a	31.62c	5235.49b	68.38b
	L40	11,076.73a	16,742.68a	2328.3b	29.12d	5665.94a	70.88a
2018–2019	RF	9809.97b	12,971.94c	2712.5a	46.17a	3161.97c	53.83c
	L30	12,230.35a	17,842.88b	2634.22a	31.94b	5612.53b	68.06b
	L40	12,593.26a	18,895.13a	2460.56b	28.08c	6301.87a	71.92a
	L50	12,969.66a	19,164.76a	2493.96b	28.70c	6195.10 a	71.30a

*DMT* dry matter translocation amount, *CDMT* contribution of pre-anthesis assimilates to grain, *DMAA* dry matter accumulation amount after anthesis, *CDMAA* contribution of dry matter accumulation amount after anthesis to grains.

Different letters indicate significant statistical differences between treatments (P < 0.05).

**Figure 4:**
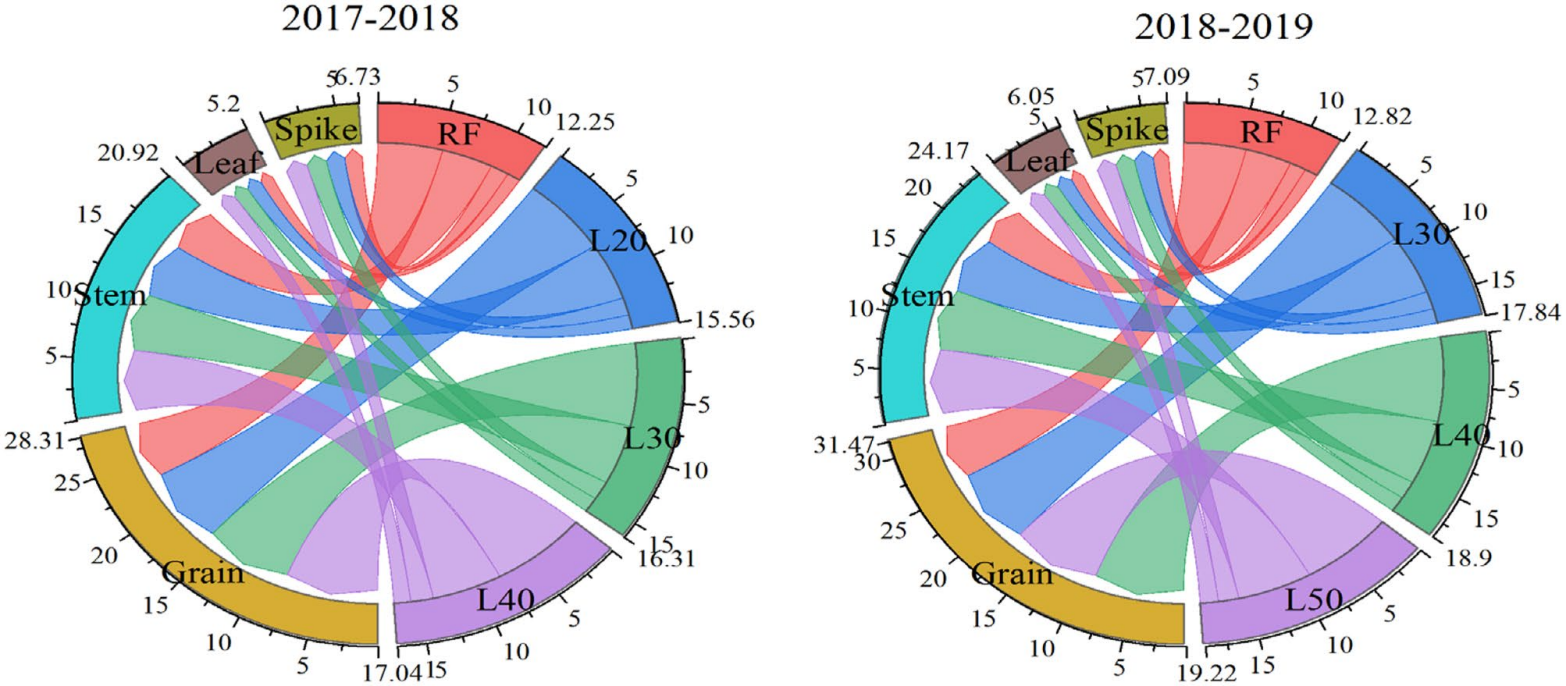
The dry matter accumulation (× 10^3^ kg ha^−1^) of different organs at maturity under different treatments in 2017–2018 and 2018–2019. In the figure, four treatments correspond to four colors, and the width of each color represents the dry matter accumulation amount.

Due to less DMM, the dry matter distribution in each organ of RF treatment was lower than that of the other treatments (Fig. 4). The dry matter accumulation of the stem, leaf, and spike both increased with the increase in border length. However, with the increase in border length, the dry matter accumulation of the grain first increased and then decreased, and the maximum value was obtained in the L40 treatment. This was mainly because the higher dry matter accumulation in the stem of the L50 treatment was not transferred to the grain.

### Grain yield and WP

**Figure 5:**
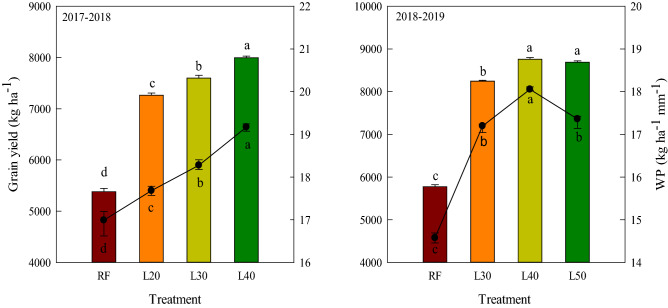
Grain yield (bars) and water productivity (circles) of different treatments in 2017–2018 and 2018–2019. The different letters above the bars and below the circles represent significant differences between the treatments at the P < 0.05.

Compared with RF, the irrigation treatments significantly improved the grain yield and WP (Fig. 5). In 2017–2018, L40 was 10.01% and 5.05% higher in grain yield, 8.43% and 4.76% higher in WP compared with L20 and L30, respectively. In 2018–2019, the grain yield of L40 and L50 was significantly higher than that of L30, and there were no significant difference in WP between L30 and L50, which were both significantly lower than that of L40.

### The correlation coefficient between grain yield and related indicators after anthesis

**Figure 6:**
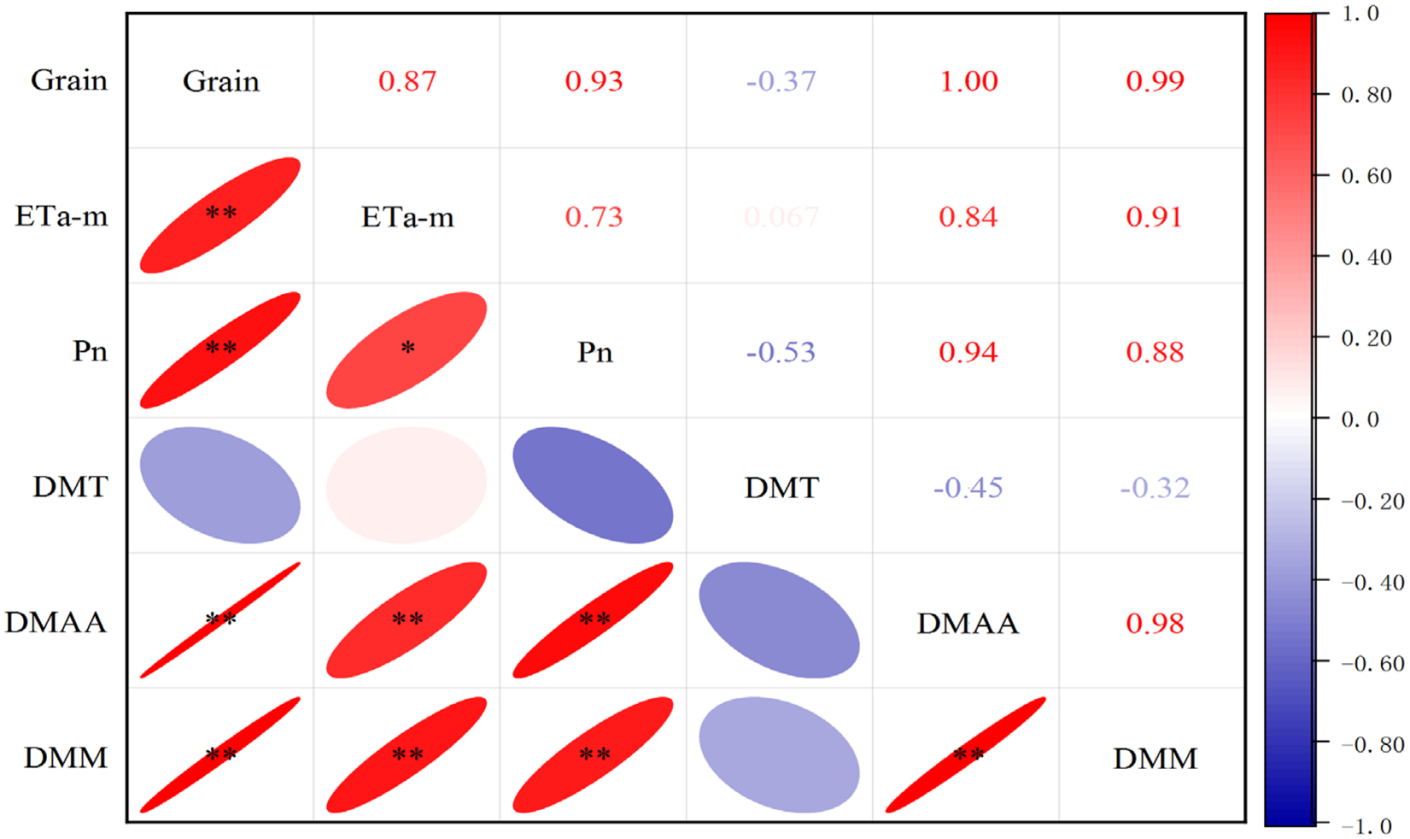
The correlation coefficient between grain yield, water consumption from anthesis to maturity (ETa-m), net photosynthesis rate (Pn), dry matter translocation amount (DMT), dry matter accumulation after anthesis (DMAA), dry matter accumulation at maturity (DMM). *,**Significant at the 0.05, 0.01 probability levels.

Figure 6 shows the results of correlation analysis based on the experimental data of the two growing seasons. The grain yield were significantly positively correlated with ETa-m, Pn, DMAA and DMM. There were also significant positive relationships between ETa-m, Pn, DMAA and DMM; however, there was no significant correlation between DMT with other indicators.

### Relationship between soil water content after anthesis irrigation with grain yield and WP

**Figure 7:**
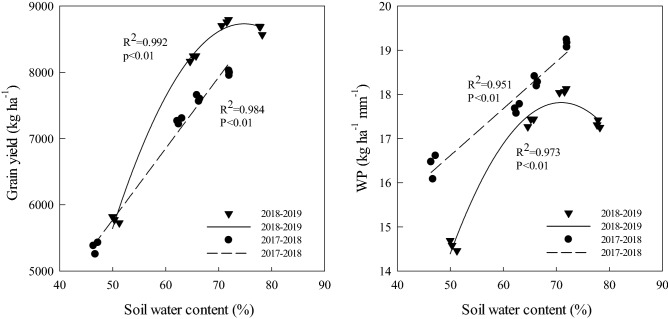
Relationship between soil water content after anthesis irrigation of 0–200 cm soil layer with grain yield and WP in 2017–2018 and 2018–2019.

The grain yield and WP have a quadratic relationship with the water content of the 0–200 cm soil layer after anthesis irrigation (Fig. 7). Within a certain range, grain yield and WP increased with the increase in soil water content. When the soil water content exceeded 71.77%, the WP began to decrease significantly, yet the grain yield did not increase significantly. When the soil water content exceeded 77.22%, the grain yield began to slowly decrease.

## Discussion

### Soil water content and water consumption under different border irrigation

Although in traditional border irrigation the irrigation is generally stopped after the waterfront reaches the end of the border, this water continues to flow toward the end of the field. Therefore, an increase in border length will not only lead to excessive irrigation but also an uneven distribution of irrigation water^19^. The results obtained in the present study corroborate these findings; the amount of irrigation water increased with border length and the soil water content of different soil layers after irrigation also increased with border length. In fact, some studies seem to confirm the inefficiency of longer border lengths. For instance, the irrigation amount of a treatment with a 180 m border length was 40 mm higher than that of a treatment with a border length of 90 m, yet the grain yield was not significantly increased^9^. In an attempt to solve this, studies have shown that an inflow cutoff of 90% can efficiently reduce the amount of irrigation water and improve the WP of crops^14^. However, even with the implementation of this method, an uneven distribution of irrigation water and a decrease in WP were still found in treatments with longer border lengths^20^. This was evident even in the results of our study, which implemented this method with significantly shorter border lengths (20–50 m).

We found a gradual increase of wheat ET associated with an increase in the amount of irrigation water, which is consistent with the findings of other studies^21^. The water consumption from jointing to anthesis of L50 treatment was 8.63% and 10.41% higher than that of L40 and L30 treatments respectively, which was the main reason for its significant increase in total water consumption compared with other treatments. This is mainly due to the high soil water content after jointing irrigation of L50 treatment, which significantly delayed the extinction of ineffective tillers after jointing, resulting in an increase in water consumption^22^. However, compared with the L40 treatment, the ET of the L30 and L20 treatments did not decrease significantly, which may be due to the increased soil water consumption and soil water evaporates^23^. This is consistent with the findings of Gao et al.^24^ that optimized irrigation can significantly improve population structure, reduce ineffective water consumption, and improve water use efficiency. Due to the lower grain yield of L20 and L30 treatments, their WP was significantly lower than that of L40 treatment, although there was a difference in the amount of irrigation water. This contrasts with the results of L50, which has a higher ET value due to higher irrigation water but no increase in grain yield, and its WP is significantly lower than that of L40.

### Photosynthetic, dry matter accumulation and grain yield under different border irrigation

Increasing DMAA or increasing the distribution of dry matter in the grain during maturity is an effective way to increase grain yield^13,25^. This was consistent with the conclusion that grain yield was significantly positively correlated with DMAA and DMM in this study. Research indicates that soil water content in the 0–50 cm soil layers is significantly affected by the amount of irrigation water during the jointing and anthesis stages^26^, which in turn can have a considerable effect on the dry matter accumulation of wheat. Zhang et al.^27^ found that when the soil water content is 70–80%, the photosynthetic rate at the grain filling stage and dry matter accumulation at maturity were 35.5% and 197.7% higher, respectively, than those when the soil water content was 40–50%. In this study, the soil water content of the 0–80 cm soil layer after irrigation of the L40 treatment was approximately 72%. The sufficient water supply of L40 treatment made the Pn of flag leaf higher by 12.36% and 21.31% respectively than that of L30 and L20 treatments at 14–28 DAA, and the DMAA and DMM were 10.25%, 17.47% and 5.1%, 9.02% higher than those of L30 and L20 treatments, respectively. However, the Pn and dry matter accumulation after anthesis in the L50 treatment with a further increase in soil water content were not significantly increased compared with the L40 treatment.

With the increase in soil water content after irrigation, grain yield increased from L20 to L40, then slowly decreased from L40 to L50. Although the water stress of the L20 and L30 treatments can increased the translocation of dry matter, they significantly reduced the accumulation of dry matter after anthesis, so the grain yield was significantly lower than that of L40. In the late anthesis stage, the L50 treatment had a higher Pn, but in the late grain filling stage, a large amount of photosynthetic products could not be transferred to the grain, resulting in a slight decrease in grain yield compared with L40, and the dry matter accumulation in stem was significantly higher than that of the other treatments. Additionally, the regression analysis of the soil water content after anthesis irrigation of the 0–200 cm soil layer with grain yield and WP also confirmed that L40 was the best irrigation border length in this experiment both terms of a high yield and water saving.

Although many new irrigation methods have been developed, the high cost and complexity of operation have resulted in low usage by farmers. Changing the border length and adjusting border field layout is a straightforward and low-cost method, which can significantly reduce irrigation water and realize uniform irrigation. Therefore, this experiment is of great significance for reducing agricultural irrigation water and maintaining sustainable agricultural development in the HPC. In addition, different soil types have a significant impact on the water infiltration rate; therefore, our next step will be to further refine the optimal border length under different soil types to better optimize traditional irrigation.

## Conclusion

Overall, our results show that under supplemental irrigation at jointing and anthesis with an inflow cutoff of 90%, the most efficient border irrigation treatment was the one with a border length of 40 m. This treatment had the highest water productivity, which was 3.98%, 4.54% and 7.94% higher than border lengths of 50 m, 30 m and 20 m, respectively. This treatment significantly increased the Pn of flag leaf after anthesis, and the DMAA increased by 17.47% and 10.25% compared with the treatments with border lengths of 20 m and 30 m. Compared with the 50 m border length treatment with a higher water input, this treatment significantly increased the distribution ratio of dry matter to the grains at maturity. Therefore, these results demonstrate that proper border irrigation can effectively save water resources by improving the soil water content and increasing the dry matter accumulation without sacrificing the grain yield of wheat.

## Data Availability

All data generated or analyzed during this study are included in this published article.
